# The Protective Effects of Curcumin on Experimental Acute Liver Lesion Induced by Intestinal Ischemia-Reperfusion through Inhibiting the Pathway of NF-*κ*B in a Rat Model

**DOI:** 10.1155/2014/191624

**Published:** 2014-08-20

**Authors:** Zhe Fan, Huirong Jing, Jihong Yao, Yang Li, Xiaowei Hu, Huizhu Shao, Gang Shen, Jiyong Pan, Fuwen Luo, Xiaofeng Tian

**Affiliations:** ^1^Department of General Surgery, The Third People's Hospital of Dalian, Dalian 116033, China; ^2^Department of General Surgery, The Second Hospital of Dalian Medical University, Dalian 116023, China; ^3^Department of Pharmacology, Dalian Medical University, Dalian 116044, China; ^4^Department of General Surgery, Liaoning Cancer Hospital and Institute, Shenyang 110042, China; ^5^Department of General Surgery, Children's Hospital of Dalian, Dalian 116000, China

## Abstract

*Objective.* In this study, we investigated the protective effect and mechanism of curcumin on a rat model of intestinal ischemia/reperfusion (I/R), which induces an acute liver lesion. *Methods.* Curcumin was injected into rats in the curcumin groups through left femoral vein. The same volume of vehicle (0.9% normal saline) was injected into sham and I/R groups. Blood and liver tissue were gathered for serological and histopathological determination. *Results.* Intestinal I/R led to severe liver injury manifested as a significant increase in serum AST and ALT levels; all of those were reduced by treatment with curcumin. Simultaneously, the activity of SOD in liver decreased after intestinal I/R, which was increased by curcumin treatment. On the other hand, curcumin reduced MPO activity of liver tissue, as well as serum IL-6 and TNF-*α* levels observably. This is in parallel with the decreased level of liver intercellular cell adhesion molecule-1 (ICAM-1) and nuclear factor-*κ*B (NF-*κ*B) expression. *Conclusion.* Our findings suggest that curcumin treatment attenuates liver lesion induced by intestinal I/R, attributable to the antioxidative and anti-inflammatory effect via inhibition of the NF-*κ*B pathway.

## 1. Introduction

Intestinal ischemia-reperfusion (I/R) injury is a serious clinical event which is always occurring in some critical conditions such as superior mesenteric artery (SMA) occlusion and thrombus, hemorrhagic shock, or small bowel transplantation. Intestinal I/R not only leads to severe intestine damage but also induced subsequent destruction of remote organs including liver, lung, and kidney as well [1–3]. Liver is the most vulnerable organ after intestinal I/R because of the theory that liver and intestine share the anatomical common pathway such as coupled vasculature [4].

Despite various research efforts over the past decades on liver lesion induced by intestinal I/R, the mechanism is still obscure and pharmacologic therapies have remained ineffective and controversial. We and others have demonstrated that many mediators are involved in the pathogenesis of intestinal I/R-induced liver injury. Among them, reactive oxygen species (ROS) by active Kupffer cells and proinflammatory cytokines [5–7] are central to this pathogenesis [4]. ROS may facilitate the liver sinusoid endothelial disruption and induced cell death. Moreover, NF-*κ*B have proved to regulate the oxidative and proinflammatory reaction. Our previous study showed that NF-*κ*B plays an important role in the pathogenesis of liver injury induced by intestinal I/R.

Curcumin is a phenolic compound derived from root stocks of the rhizomes* Curcuma longa*,* Curcuma aromatica*,* Curcuma zedoaria*, and* Acorus calamus*. Traditional Chinese medicine has used curcumin pharmacologically in cell culture and animal models. Curcumin is antineoplastic and anti-inflammatory including anti-I/R [5–7]. However, the potential protective mechanism of curcumin on acute liver lesion induced by intestinal I/R still needs to be further explored [8].

In the present study, we evaluated the effect that curcumin has on neutrophil infiltration, intracellular adhesion molecule- (ICAM-) 1 expression, and NF-*κ*B activation of liver tissues in rats. Our results indicate the feasibility of using curcumin clinically in the treatment of intestinal I/R.

## 2. Materials and Methods

### 2.1. Animals and Surgical Procedure

Male Sprague-Dawley rats weighing 190–220 g were obtained from the Animal Center of Dalian Medical University (Dalian, China; Institutional protocol number: SCXK 2008-0002) and the study was approved by the Institutional Ethics Committee. All rats were housed at a temperature of 22 ± 2°C, were kept on a 12 : 12-h photoperiod, and were all provided with food and water ad libitum. Procedures were conducted in accordance with the institutional guidelines for the care and use of laboratory animals and the study was approved by the Institutional Animal Care Committee of Dalian Medical University (Dalian, China). The rats were divided into four experimental groups randomly: sham, intestinal I/R (I/R), 1 mg/kg of curcumin treatment groups (1-cur), and 5 mg/kg of curcumin treatment groups (5-cur) (*n* = 8 in each group).

The model of rats intestinal I/R was established according to the standardized methods [9]. Briefly, after general anesthesia, a midline laparotomy was performed, and the superior mesenteric artery (SMA) was isolated gently at its root and occluded with an atraumatic microvascular clamp for 1 h and then followed by reperfusion for 2 h. The occlusion was confirmed by complete pulse cessation and the intestines became pale; then the reperfusion was confirmed by the return of pulsatile flow to the mesenteric artery and its branches.

The rats in sham group underwent surgical preparation including isolation of the superior mesenteric artery (SMA) without occlusion. The rats in the 1-cur and 5-cur groups underwent surgery with left femoral vein administration of curcumin (Shanghai Usea Biotech Company, China) after occlusion for 50 min. The same volume of 0.9% normal saline as vehicle was injected into sham and I/R groups.

The dose of curcumin administration was selected through the previous literature [10, 11] and modified from preliminary experiments. Two hours after reperfusion, blood and liver tissue samples were obtained for further analysis.

### 2.2. Liver Morphological Assessment

The isolated liver tissues were instantly collected and fixed in 10% formalin. Tissues were embedded in paraffin, cut into sections 4 microns in thickness, and stained with hematoxylin and eosin (H&E). Scores of liver pathology were evaluated by Eckhoff's reported as follows: grade 0, minimal or no evidence of injury; grade 1, mild injury consisting of cytoplasmic vacuolation and focal nuclear pyknosis; grade 2, moderate to severe injury with extensive nuclear pyknosis, cytoplasmic hypereosinophilia, loss of intercellular borders, and mild to moderate neutrophil infiltration; and grade 3, severe injury with disintegration of hepatic cords, hemorrhagic, and severe PMN infiltration. An average of 100 adjacent points on a 1-mm^2^ grid were graded for each specimen (*n* = 4) [12].

### 2.3. Measurement of Serum Aspartate Aminotransferase (AST) and Alanine Aminotransferase (ALT)

Blood samples were centrifuged (1000 g, 10 min, 4°C) and the obtained serum was then stored in a −80°C fridge. ALT and AST in the serum were measured with an OLYMPUS AU1000 automatic analyzer (AusBio Laboratories Co., Ltd., Beijing, China).

### 2.4. Liver Superoxide Dismutase (SOD) and Myeloperoxidase (MPO)

The liver tissues were harvested and homogenized immediately on ice in 5 volumes of normal saline. The homogenates were centrifuged at 1200 r/min for 10 min to remove debris. SOD and MPO were measured using an assay kit (Nanjing Jiancheng Corp., China), according to the manufacturer's recommendations.

### 2.5. Tumor Necrosis Factor- (TNF-) *α* and Interleukin- (IL-) 6 Levels

TNF-*α* and IL-6 in the serum were measured using commercially available enzyme-linked immunosorbent assay (ELISA) kits following the manufacturer's instructions (BOSTER Bio-Engineering Limited Company, Wuhan, China).

### 2.6. Immunohistochemical Analyses of ICAM-1

Liver specimens were stained by streptavidin/peroxidase immunohistochemistry technique for intercellular adhesion molecule-1 (ICAM-1) after being formalin-fixed and paraffin-embedded. The immunohistochemical tests were performed according to the manufacturer's recommendations. Four-micrometer sections were treated with 0.3% H_2_O_2_ in methanol to block endogenous peroxide activity and then incubated with the polyclonal rabbit anti-rat ICAM-1 antibody (Wuhan Boster Biological Technology Co., Ltd., Wuhan, China; both 1 : 500 dilution). Then biotinylated anti-rabbit immunoglobulin was added as a secondary antibody. The horseradish peroxidase labeled streptomycin-avidin complex was used to detect the second antibody. Finally, 3,3′-diaminobenzidine was used for color development and hematoxylin was used for counterstaining. The brown or dark brown stained cells were considered as positive. The results were evaluated semiquantitatively according to the percentage of positive cells in 5 high power fields at 400 multiple signal magnification: 0, less than 5%; 1, from 6% to 25%; 2, from 26% to 50%; 3, from 51% to 75%; 4, more than 75% [13].

### 2.7. Liver NF-*κ*B Western-Blot Assay

Nuclear protein was extracted from frozen liver tissue with a protein extraction kit reagent (KeyGEN, Nanjing, China). Each 15 ug aliquot of protein was separated by 10% SDS-PAGE gel electrophoresis. The protein was electroblotted onto NC membranes (Millipore, Bedford, MA, USA) at 9 V for 30 min. The transferred membranes were then incubated overnight at 4°C with rabbit polyclonal antibody NF-*κ*B (Santa Cruz Biotechnology, CA, USA) and Histone H3.1 (Santa Cruz Biotechnology) against rat in TBS-T (10 mmol/L Tris-HC1, pH 7.5, 150 mmol/L NaC1, 0.1% Tween-20) containing 5% nonfat milk. After being washed three times in TBS-T, the membranes were incubated for 1 h at 37°C with an anti-rabbit IgG conjugated with horseradish peroxidase. The signals were visualized using the DAB assay kit (Fuzhou Maixin Biological Technology Co., Ltd., Fuzhou, China) and documented with a gel imaging system (UVP Bioimaging System). The signals were quantitated with the Gel-Pro Analyzer Version 4.0 (Media Cybernetics, Rockville, MD, USA) [4, 14].

### 2.8. Statistical Analysis

Data were expressed as the mean ± SD. The data was processed by the statistical analysis software SPSS version 16.0 (SPSS Inc., Chicago, IL, USA). One-way analysis of variance (ANOVA) was used to determine significant differences between the groups. Multiple comparison between the groups was performed using S-N-K method. Statistical significance was accepted as *P* < 0.05.

## 3. Results

### 3.1. Curcumin Treatment Improved Histopathologic Damages of Liver after Intestinal I/R

**Figure 1:**
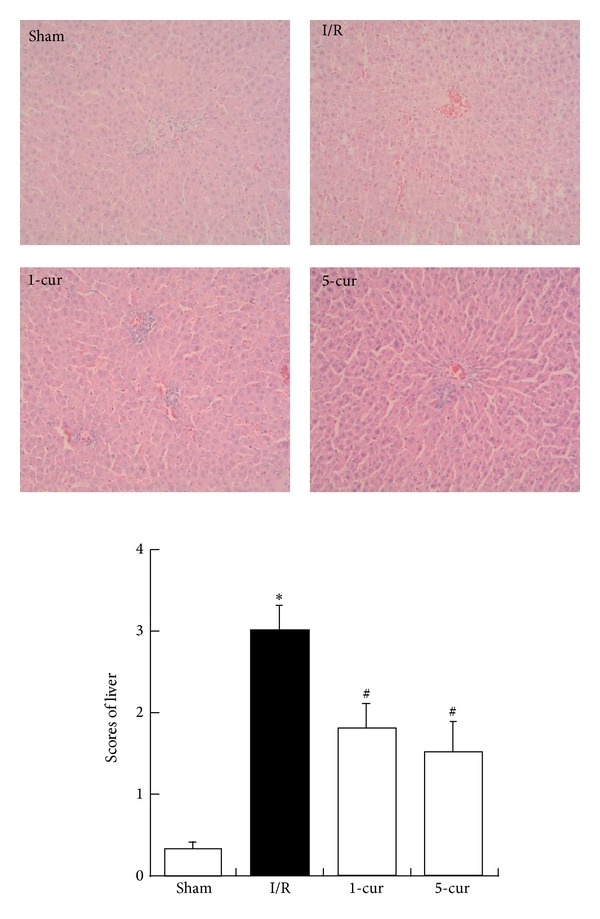
Under light microscopy at 200x magnification, histologic injury scores in groups were quantified. Results are presented as the mean ± SD, *n* = 8. *P < 0.01 versus sham groups; ^#^ *P* < 0.01 versus I/R groups.

There were no obviously morphologic changes in the liver tissues in sham group. However, significant morphologic changes were observed in I/R group (*P* < 0.01), which manifested as bleeding, neutrophil infiltration, and oedema in the liver tissues. 1-cur group and 5-cur group lead to the amelioration of liver injury markedly compared with I/R group (*P* < 0.01). Scores of liver decreased by 39% with 1-cur group and by 49% with 5-cur group compared with I/R group (Figure 1).

### 3.2. Curcumin Decreased Serum AST and ALT Levels after Intestinal I/R

**Figure 2:**
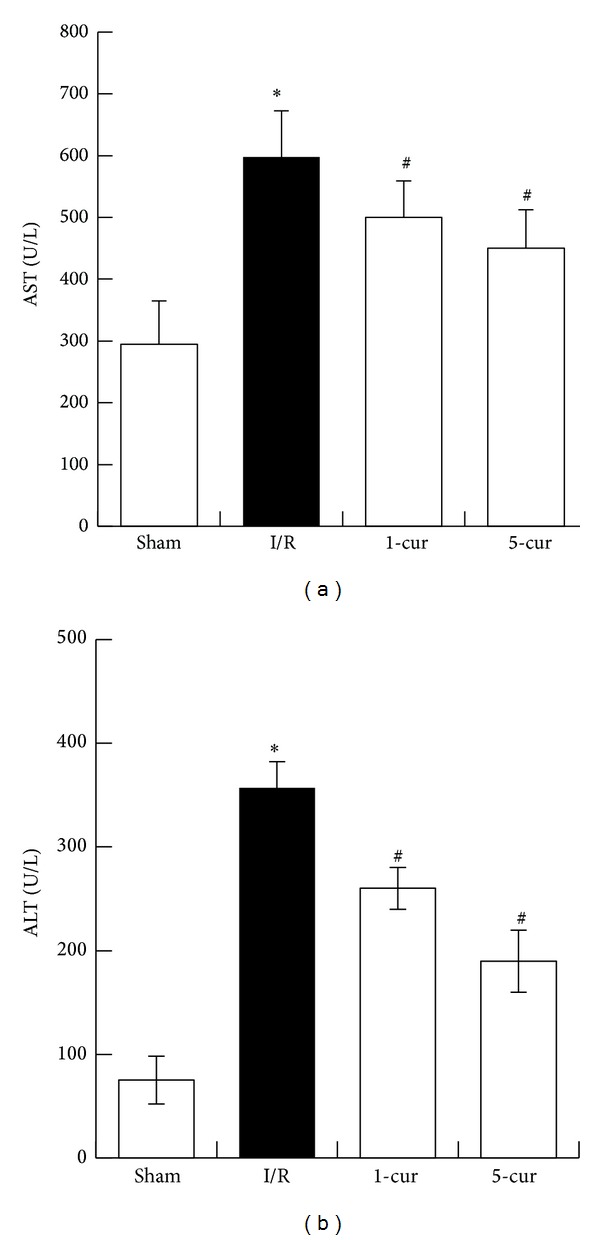
Curcumin treatment improved liver dysfunction induced by intestinal I/R. (a) AST and (b) ALT levels in different groups (mean ± SD, *n* = 8). *P < 0.01 versus sham groups; ^#^ *P* < 0.01 versus I/R groups.

Compared to the sham group, AST dramatically increased in I/R group (*P* < 0.01). Curcumin decreased AST markedly in curcumin treated groups compared with I/R group (*P* < 0.01) (Figure 2(a)). AST level decreased by 19% with 1-cur group and by 26% with 5-cur group compared with I/R group, respectively. Moreover, ALT dramatically increased in I/R group (*P* < 0.01). Curcumin decreased ALT markedly in curcumin treated groups compared with I/R group (*P* < 0.01) (Figure 2(b)). ALT level decreased by 25% with 1-cur group and by 47% with 5-cur group compared with I/R group, respectively.

### 3.3. Curcumin Restored SOD Level but Decreased MPO Level in Liver after Intestinal I/R

**Figure 3:**
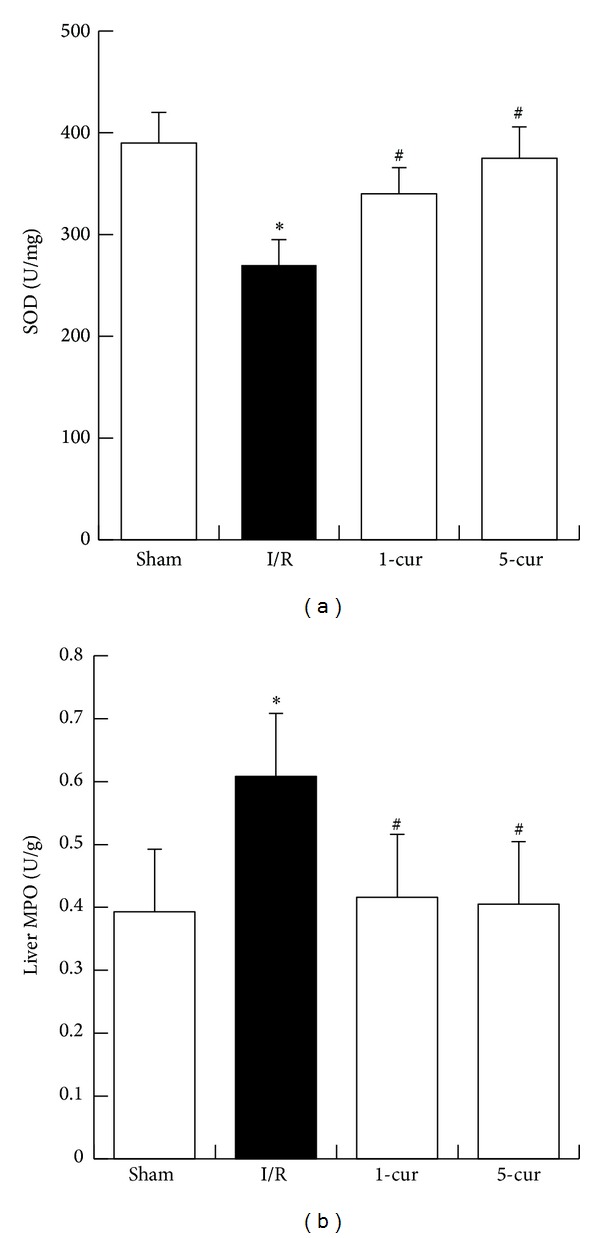
Curcumin treatment restored SOD but decreased MPO levels in liver after intestinal I/R. (a) SOD and (b) MPO levels in different groups (mean ± SD, *n* = 8). *P < 0.01 versus sham groups; ^#^ *P* < 0.01 versus I/R groups.

Compared with sham group, the level of liver SOD in I/R group significantly reduced (*P* < 0.01). However, curcumin restored SOD level markedly in curcumin treated groups compared with I/R group (*P* < 0.01, Figure 3(a)). SOD increased by 25% with 1-cur group and by 39% with 5-cur group compared with I/R group, respectively. In addition, MPO, which represents the ability of hepatic neutrophil recruitment and peroxidation, increased significantly in I/R group than in sham group after reperfusion (Figure 3(b), *P* < 0.01). However, curcumin decreased MPO level markedly in curcumin treated groups compared with I/R group (*P* < 0.01). MPO decreased by 32% with 1-cur group and by 33% with 5-cur group compared with I/R group.

### 3.4. Curcumin Decreased Serum TNF-*α* and IL-6 Levels in Liver after Intestinal I/R

**Figure 4:**
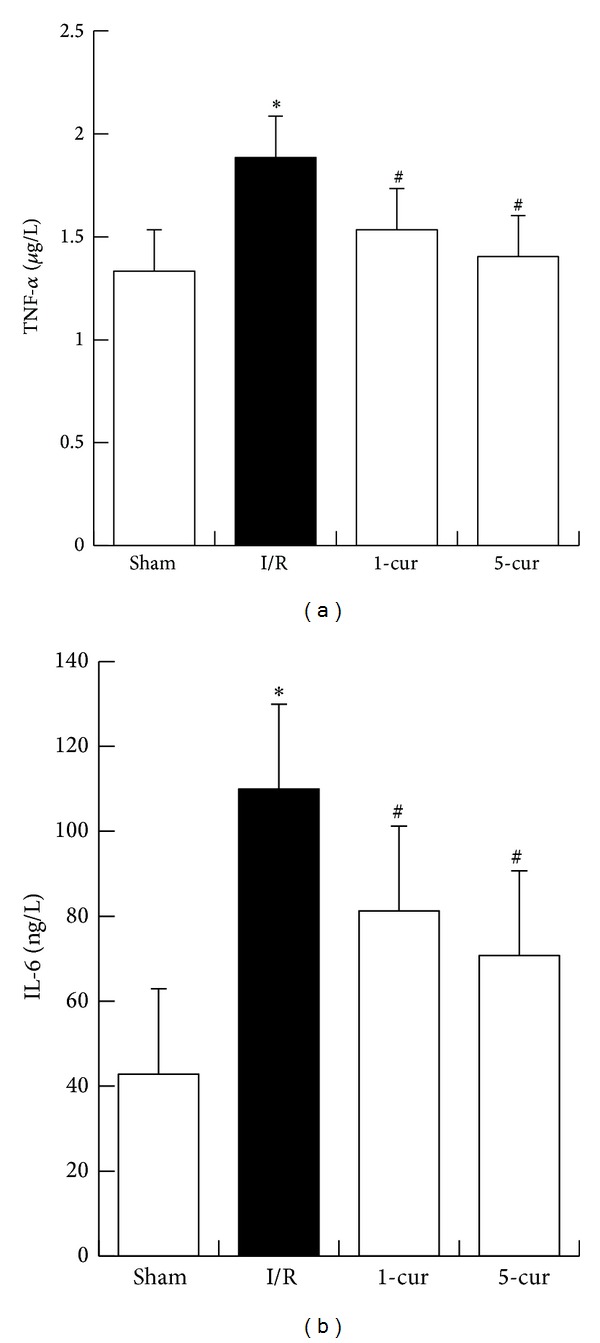
Curcumin treatment decreased serum proinflammatory levels after intestinal I/R. (a) TNF-*α* and (b) IL-6 levels in different groups (mean ± SD, *n* = 8). *P < 0.01 versus sham groups; ^#^ *P* < 0.01 versus I/R groups.

We and others have reported that TNF-*α* and IL-6 are key mediators of liver lesion after intestinal I/R [4, 14]. In this study, the two serum cytokines increased significantly after intestinal I/R compared with the sham group (*P* < 0.01, resp.). Furthermore, curcumin treatment (1-cur and 5-cur) dramatically reduced TNF-*α* levels (*P* < 0.01, resp.; Figure 4(a)). Curcumin treatment (1-cur and 5-cur) dramatically reduced IL-6 levels (*P* < 0.01, resp.; Figure 4(b)).

TNF-*α* decreased by 19% with 1-cur group and by 26% with 5-cur group compared with I/R group. IL-6 decreased by 26% with 1-cur group and by 36% with 5-cur group compared with I/R group.

### 3.5. Curcumin Decreased Expression of ICAM-1 in Liver after Intestinal I/R

**Figure 5:**
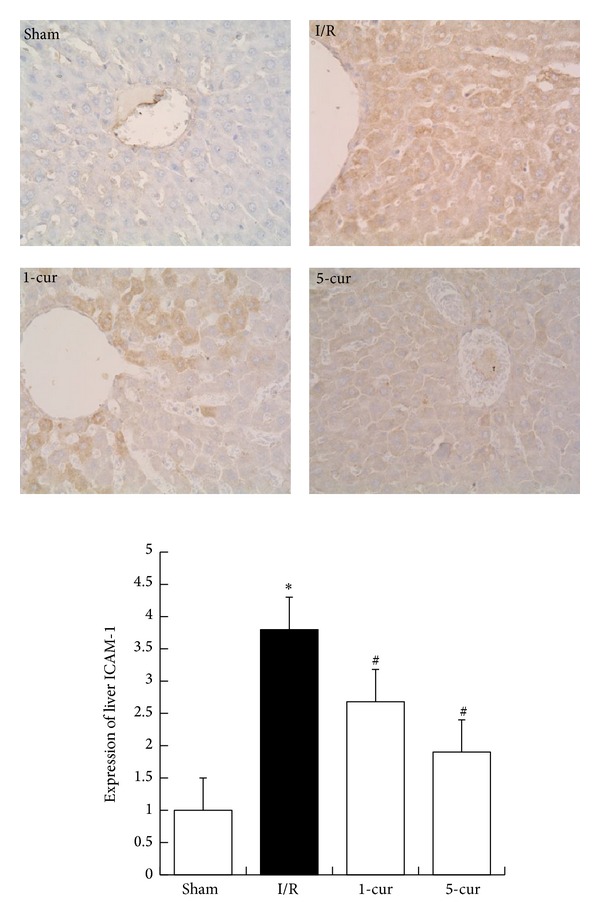
Curcumin treatment decreased ICAM-1 levels after intestinal I/R in different groups (mean ± SD, *n* = 8). *P < 0.01 versus sham groups; ^#^ *P* < 0.01 versus I/R groups.

Immunohistochemical study showed that there was weak staining of ICAM-1 observed in the sham groups. Compared with the sham group, the expression of ICAM-1 was increased significantly in I/R group (*P* < 0.01, Figure 5), while positive staining of ICAM-1 expression in the cytoplasm decreased markedly in the curcumin treatment group (*P* < 0.01, Figure 5) compared with I/R group. Expression of ICAM-1 decreased by 30% with 1-cur group and by 40% with 5-cur group compared with I/R group.

### 3.6. Curcumin Decreased Expression of NF-*κ*B in Liver after Intestinal I/R

**Figure 6:**
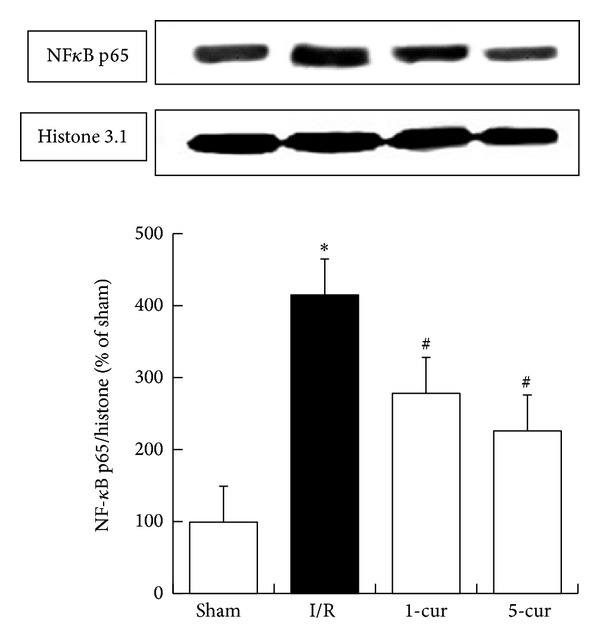
Curcumin treatment decreased NF-*κ*B levels after intestinal I/R in different groups (mean ± SD, *n* = 8). *P < 0.01 versus sham groups; ^#^ *P* < 0.01 versus I/R groups.

Compared to the Sham group, NF-*κ*B dramatically increased in I/R group (*P* < 0.01). Curcumin decreased NF-*κ*B expression markedly in curcumin treated groups compared with I/R group (*P* < 0.01, resp.; Figure 6). NF-*κ*B level decreased by 33% with 1-cur group and by 46% with 5-cur group compared with I/R group, respectively.

## 4. Discussion

Intestinal I/R is a severe event which not only leads to intestinal local dysfunction but also leads to distant organs dysfunction. Intestinal I/R often follows mesentery embolism, intestinal diseases sepsis, hemorrhagic shock, and transplantation, which was thought to be a main inducer for the occurring of multiple organ dysfunction syndromes (MODS) [15, 16]. In the remote organs, liver is the first damaged organ when the intestine suffered I/R because its vasculature is coupled with the intestinal circulation [17]. Moreover, multiple deleterious mediators including ROS, IL-6, and TNF-*α* are released and cascade associated with leukocyte adhesion and activation also contribute to liver injury [4].

Oxidative stress and inflammatory cascade are two critical pathogenesis in liver injury induced by intestinal I/R. ROS which act as a second messager will further induce inflammatory reaction. Intestinal I/R impaired the intestine barrier function. Facilitated bacteriotoxin and reactive oxygen species generate and transfer to liver which is associated with activation of NF-*κ*B pathway. NF-*κ*B inversely induces the generation of proinflammatory cytokines and eventually leads to liver injury. The circuit likes a positive feedback which makes the injury more and more deteriorated [4, 18–20]. Our present results demonstrate that curcumin (I) improved liver histopathology, (II) decreased serum AST and ALT levels, (III) decreased liver MPO but increased SOD levels, and (IV) these changes were parallel to the increased levels of liver NF-*κ*B p65, ICAM-1, serum TNF-*α*, and IL-6.

NF-*κ*B contains two subunits of the Rel family proteins and includes RelA (p65), RelB, cRel, p50, and p52 in mammalian. NF-*κ*B is also a critical transcriptional factor regulating the genes involving cell survival and immune reactions. Moreover, NF-*κ*B plays a crucial role in the cellular stress such as inflammation and intracellular levels of reactive oxygen species [18, 21]. Under common circumstance, NF-*κ*B is a heterodimer, is located in the cytoplasm, and is combined to the inhibitory unit inhibitory *κ*B (I*κ*B). The latter consists of various subtypes including I*κ*B *α*, I*κ*B *β*, I*κ*B *ε*, I*κ*B *γ*, and B-cell lymphoma 3 (Bcl3). When cells suffered stress challenge, I*κ*B *α* is phosphorylated by its kinase I*κ*B kinase (IKK) complex at serine positions 32 and 36. Subsequently, NF-*κ*B translocates to the nucleus, where it is released from the I*κ*B complex and where it functions as an activator or an inhibitor of gene transcription, such as TNF-*α*, IL-6, and ICAM-1. NF-*κ*B p65 translocated into the cellular nucleus is the pivotal procedure in this process [21].

Curcumin, a naturally occurring flavonoid compound, is derived from the rhizome of* Curcuma longa* which exhibits anti-inflammatory and antioxidative stress effects in diverse stress circumstance, including I/R, and sepsis. Having the established advantage of safe and slight adverse effects, curcumin provides a novel therapeutic approach that merits further exploration. There is a wealth of experimental evidence suggesting that multiple mechanisms of action are likely responsible for the various pharmacological effects of curcumin on stress-related signaling molecules [6–8]. These include modulation of NF-*κ*B and its target molecule, such TNF-*α* and ICAM-1, and other oxidative and antioxidative enzymes involved in I/R stress.

In the present study, rats suffered intestinal I/R-induced severe liver injury represented as morphological changes, such as bleeding, neutrophil infiltration, and oedema, as conspicuously increased serum ALT and AST levels, and as alterations in the biochemical indicators of oxidative stress in the liver. These changes were associated with the increased levels of serum TNF-*α*, IL-6, and liver NF-*κ*B p65 expression. 1-cur group and 5-cur group (1 mg/kg and 5 mg/kg) improved the morphological changes significantly. Furthermore, both 1-cur group and 5-cur group blocked the changes of the indicators above, which suggested that activation of the NF-*κ*B pathway plays an important part in the pathogenesis of intestinal I/R-induced liver injury. Curcumin which alleviated liver injury induced by intestinal I/R may occur through the NF-*κ*B pathway. The above observation is also consistent with our previous study. This result may be because curcumin inhibited TNF-*α* and IL-6 further limited activation of circulation leukocytes in the microcirculation of the liver and other tissues, which eventually led to reduced inflammation-mediated tissue injury.

ICAM-1, an element of the immunoglobulin superfamily, involved in firm adhesion and emigration of activated leukocytes in postcapillary venules, may be a crucial step in the occurring of tissue injury and organ dysfunction. Our previous studies have shown an upregulation of ICAM-1, which was regulated by NF-*κ*B following hepatic I/R. In this study, we detected liver overexpression of ICAM-1 after intestinal I/R, which was significantly decreased in the 1-cur group and 5-cur group. This indicated that curcumin could suppress leukocyte adhesion to endothelia.

MPO is an indicator that indicates peroxidation and neutrophils infiltration. SOD catalyses the superoxide anion (O_2_^∙−^) into H_2_O_2_, which is an antioxidative index used to reflect tissue function [22]. In our study, MPO was significantly increased in I/R group. Treatment with 1 mg/kg and 5 mg/kg significantly reduced the expression of MPO. This is consistent with our previous study that MG132 alleviates liver MPO significantly after intestinal I/R [21]. Nevertheless, the antioxidase SOD, which showed a contrary behavior to MPO, was significantly decreased in I/R group. Treatment with 1 mg/kg and 5 mg/kg significantly restored the expression of MPO level. This indicated that curcumin protects liver injury induced by intestinal I/R which may via maintenance balance oxidase and antioxidase. This is consistent with the studies previously reported. To our knowledge, this is the first report on the potential effect of curcumin modulated NF*κ*B and the target molecule which is involved in inflammatory and oxidative stress in intestinal I/R-induced liver injury in rats.

## 5. Conclusion

To sum up, curcumin has a beneficial effect in liver injury induced by intestinal I/R. The antioxidant effect and protective effects are closely correlated with regulation of NF*κ*B pathway. Our results suggest that appropriate natural occurring NF*κ*B inhibitor may provide a potent therapeutic treatment in liver injury induced by intestinal I/R. This study has limitations, and future studies will be performed using si-RNA and correspondence cells and gene knock-out animals both in vivoand in vitro.
